# Emerging Biomedical Applications of Sustainable Cellulose Nanocrystal-Incorporated Hydrogels: A Scoping Review

**DOI:** 10.3390/gels11090740

**Published:** 2025-09-15

**Authors:** Dinuki M. Seneviratne, Eliza J. Whiteside, Louisa C. E. Windus, Paulomi (Polly) Burey, Raelene Ward, Pratheep K. Annamalai

**Affiliations:** 1 School of Health and Medical Sciences, University of Southern Queensland, Toowoomba, QLD 4350, Australia; eliza.whiteside@unisq.edu.au (E.J.W.); louisa.windus@unisq.edu.au (L.C.E.W.); 2 Centre for Future Materials, University of Southern Queensland, Toowoomba, QLD 4350, Australia; polly.burey@unisq.edu.au; 3 Rural Clinical School, The University of Queensland, Toowoomba, QLD 4350, Australia; 4 School of Agriculture and Environmental Science, University of Southern Queensland, Toowoomba, QLD 4350, Australia; 5 First Nations Engagement, Institute for Resilient Regions, University of Southern Queensland, Toowoomba, QLD 4350, Australia; raelene.ward@unisq.edu.au; 6 School of Agriculture and Food Sustainability, University of Queensland, Brisbane, QLD 4072, Australia

**Keywords:** cellulose nanocrystals (CNCs), CNC extraction, CNC sources, CNC modification, hydrogel, biomedical applications, biocompatibility

## Abstract

Cellulose nanocrystals (CNCs), derived from renewable cellulose sources, have emerged as a versatile class of nanomaterial with exceptional mechanical strength, tuneable surface chemistry and inherent biocompatibility. In the scenario of contemporary commercial hydrogel products, which are expensive and rely on synthetic materials, the sustainable origin and unique physicochemical properties have positioned CNCs as promising sustainable functional building blocks for next-generation hydrogels in biomedical applications. Over the past decade, CNC-based hydrogels have gained momentum as soft biomaterials capable of interacting with diverse tissue types, predominantly demonstrated through in vitro cell line studies. This review critically examines the current landscape of research on biomedical applications of CNC-based hydrogels, focusing on their biomedical utility across 22 systematically screened studies. It revealed applications spanning around bone and cartilage tissue engineering, wound healing, medical implants and sensors, and drug delivery. We highlight the predominance of microcrystalline cellulose as the CNC source and sulfuric acid hydrolysis as the preferred extraction method, with several studies incorporating surface modifications to enhance functionality. Despite growing interest, there remains a lack of data for transitioning towards human clinical studies and commercialisation. Hence, this review highlights the pressing need for scalable, sustainable, and affordable CNC-based hydrogel systems that can democratise access to advanced biomedical technologies.

## 1. Introduction

Cellulose, a natural polymer widely present in plant cell walls, is a sustainable raw material for biomedical applications due to its biodegradability, non-toxicity, and renewability [1,2]. As a naturally abundant feedstock, it plays a crucial role in reducing dependence on the use of synthetic, non-renewable resources in developing biomedical materials and devices. It is chemically or enzymatically extracted from native biomass, processed, and used in various forms, namely cellulose powder, cellulose derivatives, and nanocellulose, as they offer diverse physical and chemical properties suitable for applications in drug delivery, wound healing, tissue engineering, and biosensing [3]. This scoping review has compiled all primary research studies to date that have been undertaken to assess the enhancement in functionality of potential biomedical and healthcare products by incorporating cellulose nanocrystal (CNC)-based hydrogel technologies.

Minimally processed cellulose can be used for its mechanical property potential due to its close resemblance to the native structure, where it is used in the form of microfibres, fibres, or sheets for biomedical products requiring structural integrity and flexibility. Cellulose derivatives, such as cellulose acetate, carboxymethyl cellulose, and cellulose ethers, offer greater versatility and can be processed into various forms like films, hydrogels, and fibres [4]. These derivatives are often soluble in water and other organic solvents, allowing for easy transformation into products that are stable or gel-like. Nanocellulose, which is typically produced by size reduction to the nanoscale through chemical or mechanical processes, offers access to enhanced surface functionalities, including high surface area, high specific strength, and inherent water retention properties [5]. Nanocellulose is increasingly utilised in biomedical products due to its comprehensive biocompatibility, ease of functionalisation, and potential for advanced medical applications.

Nanocellulose, depending on its source, chemical and mechanical processing and subsequent morphology, is commonly used in three forms: filament-like cellulose nanofibres (CNFs), rod-like CNCs, microbially synthesised bacterial cellulose, or nanocellulose (BC/BNC) [6]. CNFs, produced through mechanical shearing of pretreated pulp, form fibrillated networks that provide hydrogels with high elasticity and strength, ideal for dynamic wound sites. BNC, synthesised by microbial processes, offers exceptional purity, water retention, and conformability, making it particularly suited for chronic or irregularly shaped wounds [7]. Together, CNCs, CNFs, and BNC form a diverse platform of sustainable nanomaterials with distinct but complementary roles in advancing biomedical hydrogel technologies. The literature contains a substantial number of studies that have reported and reviewed the use of CNFs and BNC across a wide range of biomedical applications.

More recently, CNCs have been reported in the literature for their immense potential in advancing biomedical hydrogels. Derived from renewable biomass through sulfuric acid hydrolysis or 2,2,6,6-tetramethylpiperidine-1-oxyl (TEMPO)-oxidation, CNCs possess exceptional physicochemical properties, including a high surface area, rigidity, biocompatibility, and ease of surface modification [8]. Moreover, several toxicological studies have demonstrated the low toxicity of nanocellulose materials, particularly acid-hydrolysed CNCs, highlighting their safety across various cell types and animal models, further supporting their translational potential and reinforcing their suitability for biomedical and healthcare-related applications.

These characteristics, especially low toxicity [9,10], make CNCs a versatile candidate for enhancing hydrogel formulations. By acting as a filler and rheological modifier, CNCs can improve the mechanical strength, viscoelasticity, and processability of hydrogels, enabling advanced manufacturing techniques such as 3D printing and injectable systems [11]. Their industrial scalability, including the ability to be dried and redispersed, further underscores their practicality in biomedical applications [12].

Scoping reviews play a critical role in synthesising evidence and identifying knowledge gaps within defined research domains [13]. In recent years, several reviews have broadly addressed the biomedical potential of nanocellulose-based hydrogels, often including diverse forms such as BC, microfibrillated cellulose (MFC), and CNFs across a wide range of biomedical and pharmaceutical applications [14,15,16,17]. However, these reviews typically combine studies involving various types of nanocellulose without focusing on the distinctive properties or performance of rod-like, well-individualised CNC, also known as nanocrystalline cellulose (NCC). CNCs, particularly those derived via acid hydrolysis, enzymatic treatment, or oxidation [18,19], possess specific physicochemical characteristics and morphological uniformity that can significantly influence the behaviour of hydrogels and biomedical outcomes [20]. Despite growing interest in CNC-based nanocomposite hydrogels, no scoping analysis has yet focused exclusively on studies that include appropriate control formulations and experimentally tested CNC-enhanced biomedical systems.

To address this gap, we conducted a scoping review guided by Preferred Reporting Items for Systematic Reviews and Meta-Analyses (PRISMA) methodology. We developed a predefined protocol, constructed comprehensive keyword strings for major databases, and applied strict inclusion criteria to identify original research articles that investigated CNC-incorporated hydrogels for biomedical or healthcare applications. Only studies that performed direct comparisons between CNC-containing samples and appropriate control groups were included. Full-text screening and data extraction were conducted to ensure methodological rigour.

While this review provides a focused analysis of CNC-incorporated hydrogels with clear comparative controls and biomedical investigations, it is important to acknowledge that several relevant studies in the broader biomedical field did not meet our inclusion criteria and are therefore not covered in this analysis. However, there have been notable developments, which include (i) CNC-based hydrogels prepared with a high CNC content and minimal binder or matrix, which often demonstrate promising biomedical performance; (ii) studies employing synthetic nanocrystalline cellulose nanoribbons, which share structural similarities with CNCs yet represent a distinct nanomaterial system [21]; and (iii) investigations involving dissolved and in situ regenerated crystalline nanocellulose, where the material properties and gelation behaviour differ significantly from CNC-reinforced hydrogels [22]. Although these categories fall outside the defined scope of our review, several of them report successful biomedical outcomes and are recognised as complementary contributions to the field.

This review provides a structured analysis of the role of CNCs in enhancing hydrogel systems, with particular emphasis on the source, preparation, surface modification, and biomedical performance outcomes of CNC-integrated hydrogels.

## 2. Results and Discussion

**Figure 1:**
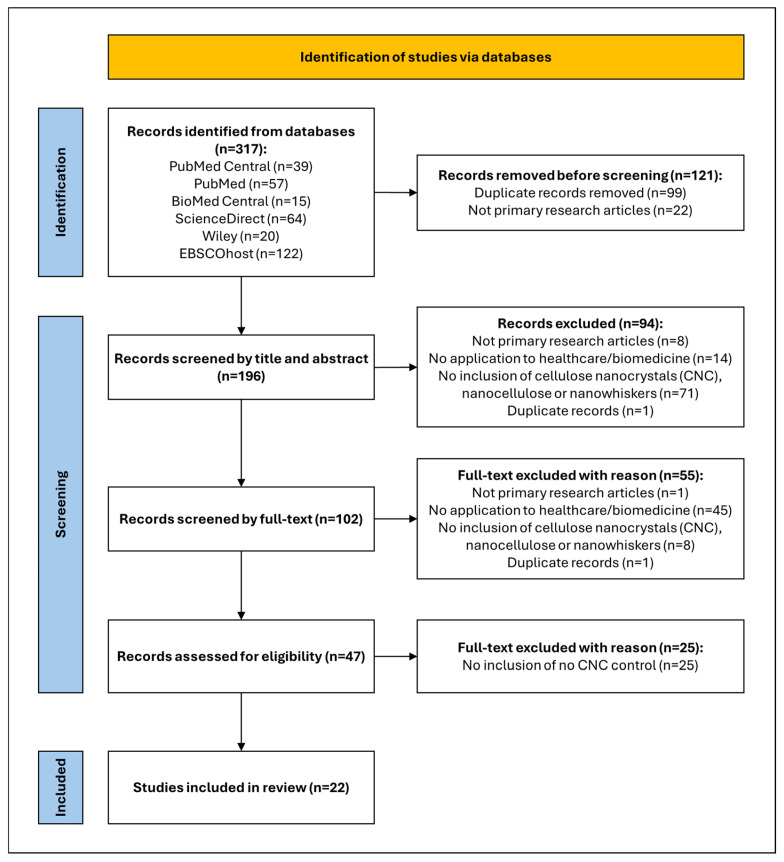
PRISMA flow diagram of the search strategy.

The scoping review searched across six databases and retrieved 317 articles (Figure 1), where 22 review articles and 99 duplicate records were removed before screening. The title and abstract screening were conducted on 196 studies, where 94 records were excluded due to not meeting the inclusion criteria. Then, 102 studies were screened during the full-text screening stage, which excluded 55 studies and included the remaining 47 studies. When these studies were further screened for the appropriate controls, 25 studies were excluded, and 22 studies were included in the scoping review.

**Figure 2:**
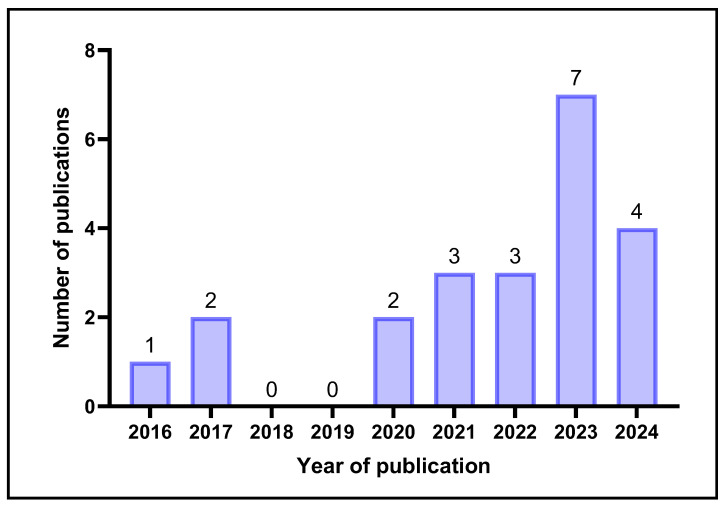
Overview of the number of publications including the search terms in their title and/or abstract per year from 2016 to 2024 (created by D.M. Seneviratne using GraphPad Prism 10.5.0).

The year of publication of the included studies commenced in 2016, where there were no publications before 2016 or 2018–2019. The results displayed in Figure 2 highlight that research into the utilisation of CNC-based products with potential biomedical applications has started to advance since 2022, with the highest number of studies published in 2023. However, there is still a lack of clinical trial results reported in the literature on the safety and efficacy of CNC-incorporated products in healthcare applications.

### 2.1. CNC Sources, Extraction, and Modifications

Some of the included studies extracted CNCs from various sources, while other studies purchased commercially available CNC products. These were further modified before the incorporation of CNCs into hydrogels. These details are collectively outlined in Table 1.

**Figure 3:**
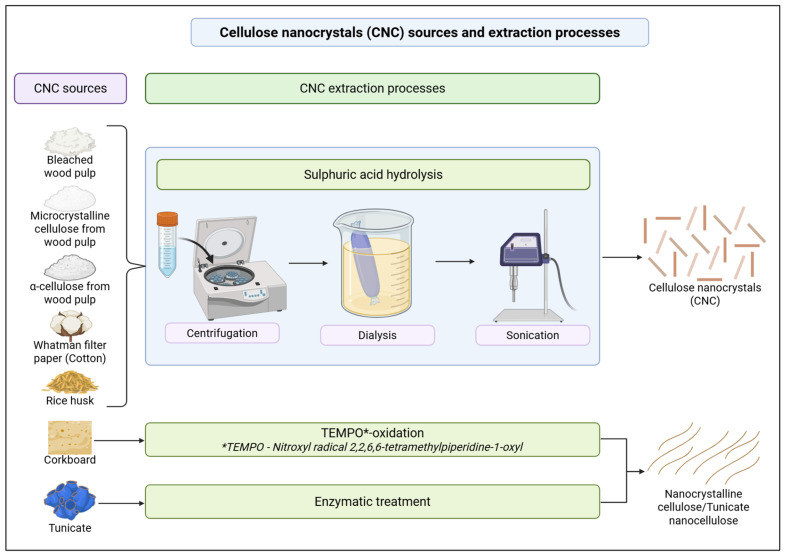
Extraction processes of cellulose nanocrystals (CNC) from various sources (created by D.M. Seneviratne using BioRender (https://app.biorender.com/ accessed on 27 April 2025).

The findings outlined in Table 1. highlight that the studies primarily purchased CNCs or microcrystalline cellulose (MCC) commercially and used Whatman filter paper as the cotton source for CNC extractions. While some studies have not specified their CNC sources [42,43], other studies have stated that CNCs were sourced from rice husk [28], corkboard [41] or commercially purchased α-cellulose [35]. Most of the studies follow the sulphuric acid hydrolysis technique for the extraction of CNCs from non-CNC sources, while one study used TEMPO-oxidation and mild disintegration in water [41]. TEMPO-originated nanocrystalline cellulose and enzymatically pretreated tunicate nanocellulose are included in this study, as they are longer nanocrystalline cellulose than the regular acid-hydrolysed CNCs. Additionally, grafting has been reported as the main CNC modification, where thiol, L-cysteine [23], cationic [25], magnetic [37] and dialdehyde [26] grafting on CNCs have been conducted. CNCs have also displayed the ability to be loaded with glucose oxidase as a model enzyme for analysing glucose levels in sweat [24] and silver nanoparticles to impart antimicrobial activity [38]. The CNC extraction processes are further detailed in Figure 3.

### 2.2. Biomedical Applications

Due to the biocompatible nature of CNC-based hydrogels, they have been widely explored as a potential material for biomedical applications. These hydrogels consistently display their positive contributions towards maintaining cell morphology, adhesion and viability to promote cell migration and proliferation. These aspects of biocompatibility are deemed crucial for applications in tissue engineering, drug delivery and biosensing.

CNC-based hydrogels can be utilised as hybrid hydrogel scaffolds [35], where they are not limited to standalone applications. These hybrid hydrogel scaffolds combine the strength of their core materials/compounds, resulting in improved mechanical properties and functionality. This versatility opens new avenues for developing advanced biomaterials tailored to specific biomedical needs. Their promising properties and ability to mimic the extracellular matrix and support cell growth make them invaluable for research and development in regenerative medicine.

#### 2.2.1. Tissue Engineering

CNC-incorporated hydrogels display favourable properties towards cell behaviour and other factors related to biocompatibility, including biodegradation, minimising inflammatory responses and blood compatibility. Due to these factors, CNC-based hydrogels are widely utilised in various tissue engineering applications, including bone and cartilage, as they are versatile and ideal for developing scaffolds that mimic the structure and functions of the natural extracellular matrix of various tissues. Bone and cartilage tissue engineering applications explored in the included studies are outlined in Table 2.

##### Bone Repair and Regeneration

Hydrogels have displayed great potential to be utilised in hard tissue engineering, particularly in bone repair and regeneration. This has been mainly attributed to the mimicking of a natural extracellular matrix by hydrogels that can be calcified and ossified towards developing bone. The hydrogels have also displayed their ability to be functionalised with various growth factors and other bioactive molecules, which have contributed towards enhancing bone regeneration capacity. CNC-based hydrogels have been investigated by three of the included studies [27,29,43] in bone tissue engineering, and their findings are summarised in Table 2.

**Figure 4:**
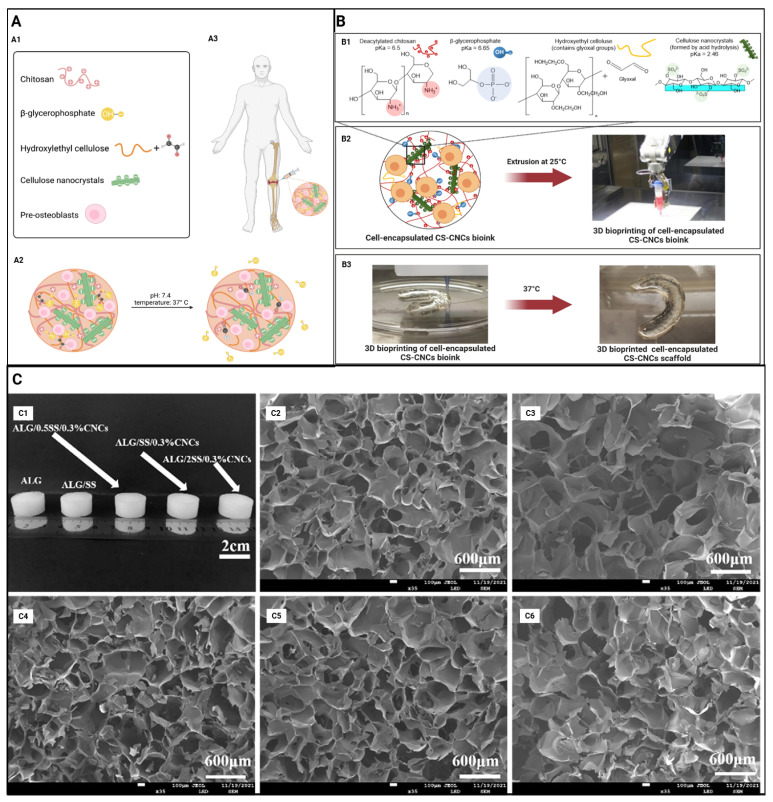
(**A**) Schematic illustration of an injectable hydrogel system. (**A1**) Formulation components for an injectable hydrogel system. (**A2**) Crosslinking mechanisms under physiological conditions. (**A3**) Desired site of injection. Adapted from reference [43]. (**B**) Schematic illustration of the 3D bioprinting process. (**B1**) Bioink formulation consisting of CS, BGP, HEC, and CNCs seeded with cells. (**B2**) Cell-encapsulated bioink was loaded into 3D-bioprinter cartridges and bioprinted onto a cell-culture glass coverslip with cartridge temperature controlled at 25 °C. (**B3**) A 3D-bioprinted scaffold of a patient-derived knee meniscus using the CS–CNC placebo bioink. The bioprinted scaffold was spontaneously gelled on a glass printing plate by temperature stimulation at 37 °C. Part of the figure was reproduced from [45]. Adapted from reference [27]. (**C**) (**C1**) Physical images of ALG hydrogels, ALG/SS, ALG/0.5SS/0.3%CNCs, ALG/SS/0.3%CNCs, and ALG/2SS/0.3%CNCs composite hydrogels; SEM images of (**C2**) ALG hydrogels, (**C3**) ALG/SS, (**C4**) ALG/0.5SS/0.3%CNCs, (**C5**) ALG/SS/0.3%CNCs, and (**C6**) ALG/2SS/0.3%CNCs composite hydrogels. ALG/SS/CNCS, alginate/sericin/cellulose nanocrystalline; SEM, scanning electron microscope. Adapted from reference [29].

The effects of various printing and manufacturing methods of hydrogels on cell viability have been explored by including studies where it has been reported that cell-laden injectable CNC-based hydrogels and cast or 3D-printed hydrogels [27] did not significantly affect the viability of MC3T3-E1 cells. CNC-based hydrogels (Figure 4A) have been signified as biocompatible due to their capability of promoting cell proliferation, as observed with MC3T3-E1 [43]. This is due to CNC incorporation mimicking the 3D environment required for cell growth and the enhanced mechanical strength of the hydrogel [29].

The incorporation of CNCs into hydrogels has been used in printing biomimetic constructs (Figure 4B) to match the bone mineral density and osteogenic differentiation of bone tissue in effectively repairing any bone defects and in bone tissue engineering [27]. Collagen formation in the extracellular matrix (ECM) and calcium mineralisation are two other measures used by studies to investigate the bone regeneration capacity of CNC-incorporated hydrogels (Table 2). In the presence of CNCs, a significant increase in collagen formation and calcium deposition in hydrogels was observed by days 14 and 21. The authors observed a high percentage of ECM area formation and an increase in calcium deposition with increased CNC incorporation [43]. Collectively, these studies indicate that accelerated early preosteoblast differentiation, mineralisation of the ECM and maturation of bones are promoted by CNC-driven collagen formation and calcium deposition.

Alkaline phosphatase enzyme (ALP) activity has been widely utilised by many studies to investigate the effects on early-stage osteogenic differentiation, where a high ALP activity is indicative of a rapid osteogenic process. The observed increase in ALP activity within the CNC-based hydrogels reflects that the incorporation of CNCs leads to improved osteogenic differentiation. These findings may be partly attributed to the production of a thick apatite particle layer in CNC-incorporated hydrogels (Figure 4C) due to enhanced biomineralisation, which was not observed in non-CNC-incorporated hydrogels [29]. Moreover, CNC concentration has been reported to be directly proportional to ALP activity [43], enhancing the efficacy of bone healing through improved osteogenesis.

##### Cartilage Repair

Hydrogels exhibit mechanical properties similar to native cartilage, providing adequate support and flexibility to mimic the biomechanical nature of cartilage [46].

**Figure 5:**
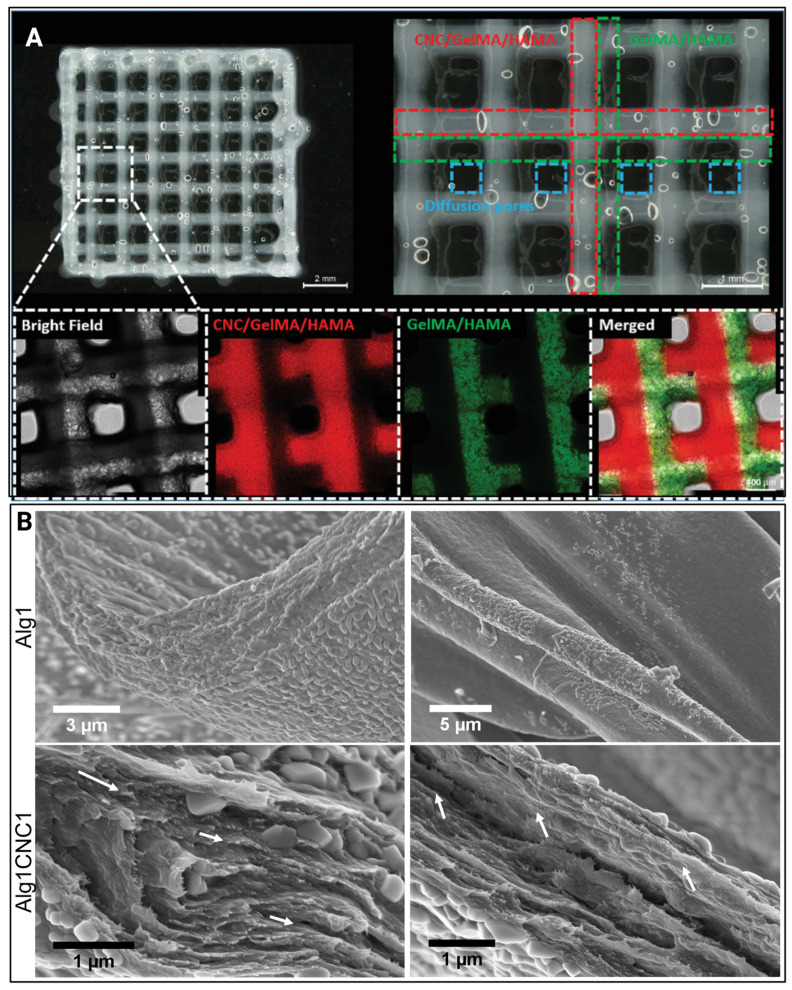
(**A**) Optical and confocal microscopic observation of the hybrid printed construct. The structure fabricated by the CNC-reinforced ink and GelMA/HAMA ink was defined in the optical microscopic image by red dotted lines and green dotted lines, respectively. The pores formed were defined by the blue dotted lines. Fluorescence in the confocal images comes from the fluorescence-labelled GelMA (red: rhodamine-labelled GelMA; green: FITC-labelled GelMA) in the CNC-reinforced ink or GelMA/HAMA ink. The scale bars are indicated. Adapted from reference [35]. (**B**) Scanning electron microscope (SEM) images of Alg1 and AlgCNC1 filaments formed via extrusion-based 3D printing. Images taken show the cross-sections of the filaments from lyophilised 3D-printed constructs at magnifications of (from top left to bottom right) 45k×, 25k×, 162k×, and 133k×. Arrows indicate the preferential local directionality of CNCs. Adapted from reference [39].

Current studies have utilised hydrogels in cartilage soft tissue engineering due to their ability to withstand mechanical stresses experienced by native cartilage [47]. In addition to the hydrogels being able to facilitate and maintain the regeneration of functional cartilage, they can integrate with the cartilage microenvironment without inducing any adverse effects [48]. One of the included studies has explored the potential of CNC-based hydrogels in cartilage engineering [35], and its findings are summarised in Table 2. CNC-incorporated hydrogels have been implicated in cartilage repair and regeneration by promoting cellular stability and growth [35]. Hybrid-printed CNC hydrogels (Figure 5A) have been shown to maintain good chondrogenic mouse ATDC5 cell viability [35], while bioprinting hydrogels (Figure 5B) has been reported to maintain better human-derived TC28a2 immortalised chondrocyte viability (94%) compared to manually encapsulating cells (>70%) [39]. These findings suggest that incorporating CNCs into hydrogels can assist in initiating chondrogenic activity.

#### 2.2.2. Wound Healing and Repair

Nanocomposite CNC-based hydrogels have been reported to maintain and promote cell viability and proliferation in neonatal and adult human dermal fibroblasts (HDFs) [30]. CNC-incorporated hydrogels pose as suitable candidates for skin tissue engineering, where one of the included studies has explored the ability of these hydrogels to be utilised in wound healing.

Wound environments involve heightened inflammatory responses and damaged epithelial linings, where CNC-based hydrogels have been shown to promote cell migration and proliferation while also minimising inflammatory responses [49]. There is a lack of studies that investigate the potential of CNC-incorporated hydrogels in wound healing, repair and regeneration, and the findings of the included study are summarised in Table 3.

The findings of the included study (Table 3) display promising effects of CNC-based hydrogels towards wound healing and repair processes.

**Figure 6:**
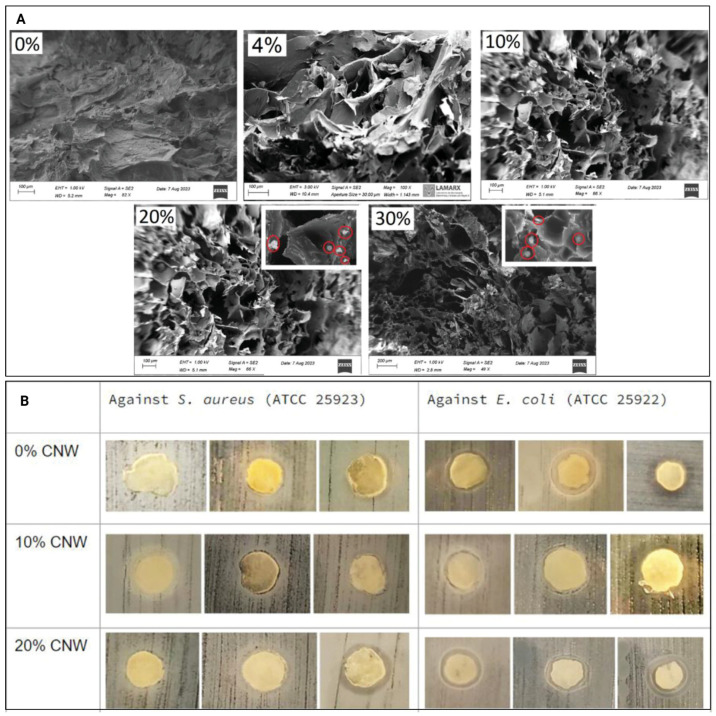
(**A**) Scanning electron microscope (SEM) images of hydrogels with 0%, 4%, 10%, 20%, and 30% CNW. Some CNW aggregates are marked in red circles. (**B**) Antibacterial test results against *Staphylococcus aureus* and *Escherichia coli* in hydrogels with 0%, 10%, and 20% CNW, showing the inhibition halo around the hydrogel samples. Adapted from reference [30].

The exposure of human dermal fibroblasts to CNC-based hydrogels (Figure 6A) displayed increasing cell numbers over seven days, indicating that these hydrogels promote cell proliferation. Thus, CNC-based hydrogels were reported to display minimal cytotoxicity, with 10% CNC hydrogels being non-toxic towards adult human dermal fibroblasts and 20% CNC hydrogels displaying only weak cytotoxicity [30]. However, as only one included study has investigated the effects of CNC-based hydrogels compared to hydrogels without CNCs on wound healing, there is a need to conduct further research on wound healing and repair using CNC-based hydrogels to support these findings.

#### 2.2.3. Medical Implants and Sensors

CNC-incorporated hydrogels have been explored for their applicability as medical implants [36,41,44] and also biosensors [24]. The findings of the included studies are summarised in Table 4.

Two of the included studies have explored the ability of these hydrogels in cardiac valve engineering [36] and endothelial-to-mesenchymal transition inhibition [41] due to CNC-based hydrogels promoting cell–cell interactions of neighbouring cardiac cells.

##### Cardiac Valve Regeneration

**Figure 7:**
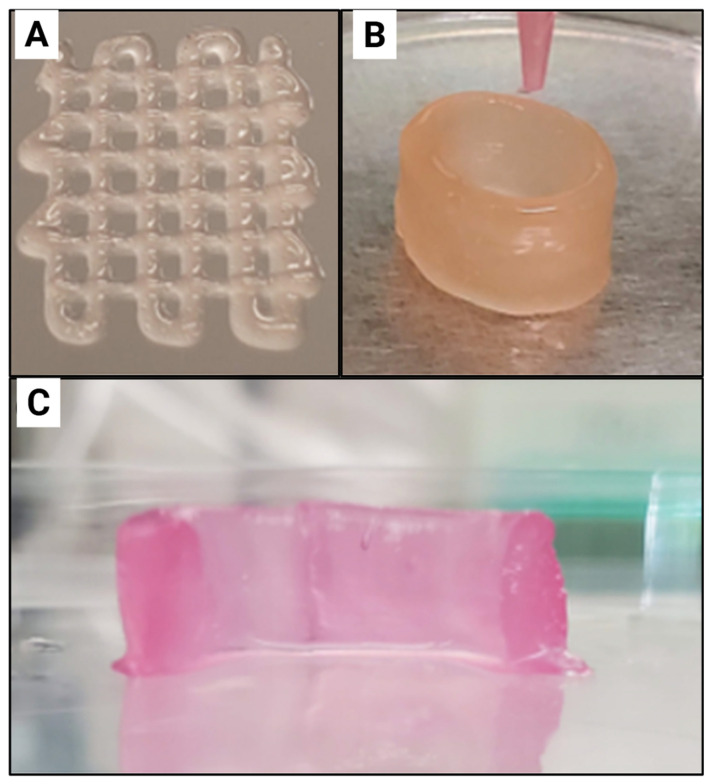
Bioprinting of mNG hydrogels. (**A**) a grid pattern and (**B**) a tubular construct (d = 20 mm, h = 15 mm, t = 3 mm). (**C**) The cross-section of the hydrogel after 7 days in culture shows robustness of the printed structure. Adapted from reference [36].

CNC-incorporated hydrogels (Figure 7A,B) have been proposed to be suitable candidates for engineering heart valves [36].

Based on an alizarin red stain of the hydrogels, the authors demonstrated that CNC incorporation is suitable for heart valve engineering since it reduces the osteogenic potential of the hydrogel when targeting cardiac valve regeneration. This was observed as a reduction in red-stained regions, indicating reduced calcium deposition and osteogenic differentiation of human adipose-derived mesenchymal stem cells. This can be attributed to these cells having a lower mineralisation capacity. Additionally, this study stated that the initially developed chondrogenic phenotype, which later transforms into a fibroblastic phenotype, mimics phenotypic properties observed in heart valve spongiosa. The CNC-incorporated hydrogels display promising results in being utilised to engineer the fibrosa layers as well, due to these hydrogels promoting GAG deposition and reducing the tendency for calcification [36]. Therefore, it can be supported that these hydrogels have the potential to be utilised in engineering multiple layers of the heart valve (Figure 7C).

Similarly, another study observed that CNC-incorporated hydrogels promoted the chondrogenic phenotype of the human adipose-derived mesenchymal stem cells, where a remodelling phase was reported after 14 days with increased matrix metalloproteinase (MMP) 1 levels and decreased MMP2 and hydroxyproline levels. The induction of human adipose-derived mesenchymal stem cell chondrogenic differentiation and its maintenance were marked by upregulated vimentin, downregulated SMA, and increased levels of SRY-box transcription factor 9 (Sox9) and aggrecan (ACAN) chondrogenic/spongiosa gene expression markers [36].

Hydrogels with the incorporation of TEMPO-oxidised CNC-based hydrogels displayed increased human adipose-derived mesenchymal stem cell spreading, signifying a direct correlation between the amount of incorporated CNCs in a hydrogel and the metabolic activity of cells [36]. Bioprinting of these hydrogels has been reported to further enhance cell viability and maintain cell morphology of human-derived TC28a2 immortalised chondrocyte cells, achieving 94% cell viability compared to >70% with manual encapsulation in adipocytes in a Balb/C mouse model [44].

##### Extravascular Stent

**Figure 8:**
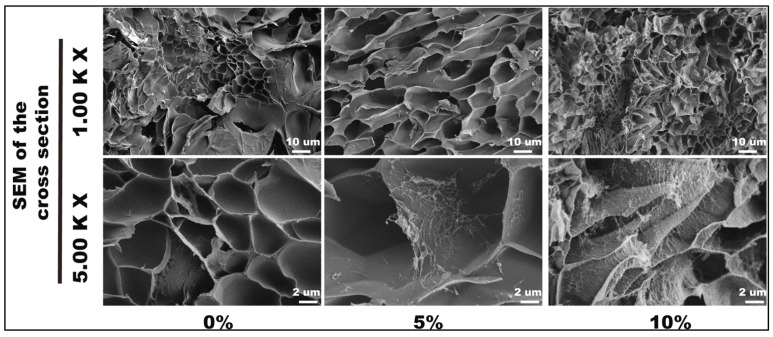
Scanning electron microscopy (SEM) of nanocellulose (NC) hydrogels with different nanocellulose (NC) concentrations. Adapted from reference [41].

A recent study explored the use of CNC-incorporated hydrogels (Figure 8) to develop extravascular stents, aimed at reducing vein graft failure [41]. These hydrogel-based stents were implanted in a rat autologous jugular vein-carotid artery graft model to optimise their clinical application.

The authors observed that these stents activated autophagy and inhibited inflammation, thereby preventing endothelial-to-mesenchymal transition (EndMT) [41]. Double staining of LC3, an autophagy core protein, and Twist, an EndMT core protein, revealed that the stents increased LC3 expression and decreased Twist expression, supporting autophagy activation to inhibit EndMT [41]. Overall, these rats were seen to display a decrease in flow rate and an increase in patency due to the inhibition of restenosis. H&E staining further confirmed these findings by showing a reduction in intimal hyperplasia and decreased intimal and wall thicknesses. Interleukin-1β expression, which is key in inducing EndMT, was also downregulated by CNC-incorporated hydrogel-based stents [41]. Lowered expressions of SMA, along with other hallmarks of EndMT, including Slug, Snail and vimentin, and increased CD31 expression were supportive of the inhibition of EndMT by the stents [41].

The retention of original cell morphology was further supported by the absence of morphological changes in main organs, including the heart, liver, spleen, lungs and kidneys, when a CNC-based hydrogel stent was locally embedded in mice [41]. This study highlighted that CNC-based hydrogels promote good cell viability and cell growth in human umbilical vein endothelial cells (HUVECs) [41]. A stent that contained 10% CNCs that was embedded in mice was observed to trigger macrophage infiltration in local tissue after three days, as signified by increased CD68, CD206 and iNOS levels [41]. However, these levels were reduced after seven days, where there was no significant difference between the CNC-based stent and the control hydrogel, supporting the biocompatibility of the stent. These results were further supported by the lack of apparent changes in organs, including the lung, heart, kidney, spleen, and liver, and changes in pro- and anti-inflammatory cytokine levels measured in blood or spleen samples [41]. Additionally, as the incorporated levels of CNCs increased, the weight lost from the hydrogels decreased, indicating a slower degradation rate. Hydrogels with a 10% CNC concentration displayed the slowest in vivo total degradation rate of 7.8 ± 0.01% after 28 days [41].

##### Autologous Fat Grafting

Autologous fat grafting is frequently employed to enhance or reconstruct areas requiring volume maintenance or replacement, with adipose tissue being widely utilised in this procedure. However, hydrogels are being presented as an emerging platform for autologous fat grafting due to their ability to mimic biological extracellular matrices while being biocompatible and promoting cell–cell interactions. Researchers are incorporating other components into these hydrogels to tailor their behaviour and improve the stability of these grafts and the ability to preserve the natural environment. Only one of the included studies has explored CNC-based hydrogels as a candidate in autologous fat grafting for soft tissue reconstructions [44], and the findings are summarised in Table 4.

##### Glucose Level Sensor

Biosensors can detect environmental changes as stimuli and convert them into reflective, measurable electrical or optical signals, a valuable feature in biomedical applications. Their ability to continuously monitor fluctuations in dynamic environments and produce user-comprehensible signals makes them particularly useful. Therefore, these biosensors can detect various external or internal stimuli, such as chemical, mechanical, or biological, and trigger signals through pressure changes, light production, or electrical responses. While hydrogels are currently used to design biosensors, CNCs have demonstrated biocompatible characteristics and versatility for various biomedical applications. Consequently, CNC-incorporated hydrogels have been investigated as biosensors for detecting glucose levels in sweat [24].

**Figure 9:**
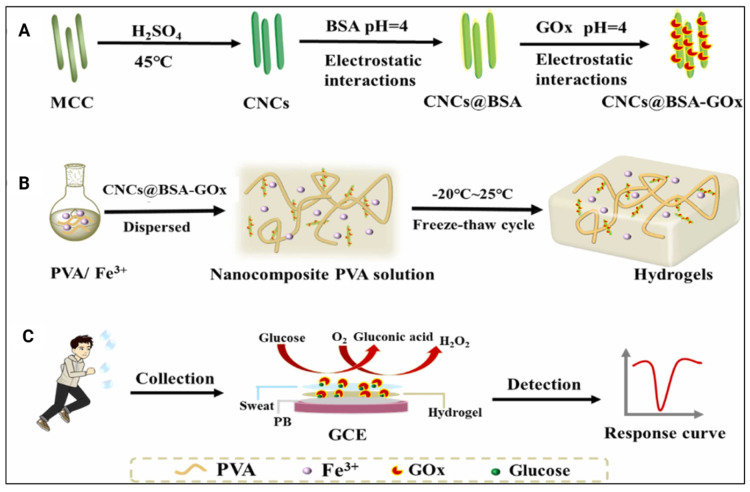
The schematic illustration of the synthesis of (**A**) CNCs@BSA-GOx and (**B**) nanocomposite hydrogels. (**C**) Schematic diagram of the hydrogel applied to sweat glucose detection. Adapted from reference [24].

One of the included studies designed hydrogels with CNCs that are loaded with glucose oxidase (Figure 9A,B) to determine glucose levels in human sweat using differential pulse voltammetry (Table 4). The findings reported that these hydrogels could detect various glucose levels in human sweat by showing corresponding changes in current values (Figure 9C). Additionally, these hydrogels demonstrated excellent adhesion to various surfaces, supporting their potential use as glucose sensors in sweat [24]. Overall, these studies highlight that incorporating CNCs in hydrogels enhances the stability and sensitivity of wearable biosensors while improving their mechanical functionalities, suggesting their vast potential for a range of biosensing applications.

#### 2.2.4. Drug Loading, Retention, and Release

Hydrogels have emerged as a favourable platform for controlled and sustained drug delivery due to the various mechanical and physical properties they possess. The hydrophilic nature of these hydrogels has enabled researchers to manipulate the molecular network by incorporating other components, thereby fine-tuning their release kinetics and integrating them with biological microenvironments. These qualities have improved the potential of hydrogels to be tailored for the delivery of various drugs and the co-delivery of multiple therapeutic agents to achieve enhanced therapeutic efficiency. The controlled and targeted manner of drug delivery could distribute drugs for an extended period [23], while hydrogels could also be used as injectable drug carriers [25]. Additionally, these hydrogels could be used as patches to navigate drug delivery using magnetism and pH stimulation [37] to reinforce controlled [28] and sustained drug delivery [31]. Any of the included studies that investigated various drug kinetics and their details are summarised in Table 5.

##### Anti-Inflammatory Drug Release

The anti-inflammatory drug release capability of the CNC-based hydrogels has been modelled by loading ibuprofen in two of the included studies [32,40].

**Figure 10:**
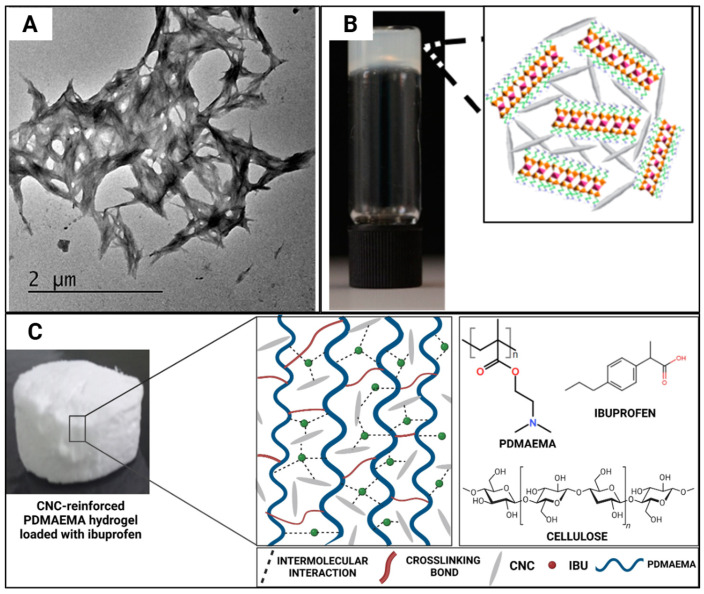
(**A**) Unstained transmission electron microscope (TEM) image of cellulose nanocrystal (CNC)-organoclay hydrogel; (**B**) photograph showing self-supported CNC:organoclay (1:0.13) colloidal nanocomposite hydrogel, inset showing schematic illustration of cross-linked network formed by non-covalent interactions between CNC (grey) and exfoliated organoclay sheets. Adapted from reference [32]. (**C**) Appearance of hydrogels post-freeze-drying and schematic representation of their hypothesised structural arrangement and ibuprofen entrapment through intermolecular interactions. The chemical structures of the polymer matrix (PDMAEMA—poly(2-(Dimethylamino)ethyl methacrylate)), nanofillers (cellulose nanocrystals—CNC), and drug (ibuprofen—IBU) are displayed on the right. Created using Biorender. Adapted from reference [40].

Ibuprofen aqueous solution (10 wt%) displayed a controlled release over 6 h, and a 10% slower release rate was displayed in CNC-incorporated hydrogels (Figure 10A,B), since this is dependent on both charge-mediated and diffusion-limited processes [32]. In another study where CNCs were incorporated at 4, 8, and 12% *w*/*w* concentrations (Figure 10C), the hydrogels gained weight instead of losing weight, and the authors stated that the presence of CNCs did not affect the stability of the hydrogels [40]. The incorporation of CNCs increases the drug loading capacity since it enhances the swelling capacity of the hydrogel, which was seen when ibuprofen (200 mg/mL) [40] was loaded.

##### Antimicrobial Drug Release

Hydrogels have been extensively researched for their antimicrobial properties, where recent studies have highlighted that hydrogels are capable of hindering the growth of microbial populations via the controlled release of encapsulated biomolecules. The included studies have used various microbial populations to investigate the antimicrobial activity of CNC-incorporated hydrogels towards these microbial populations. The explored microbial populations and the measured activities of the included studies are summarised in Table 5.

**Figure 11:**
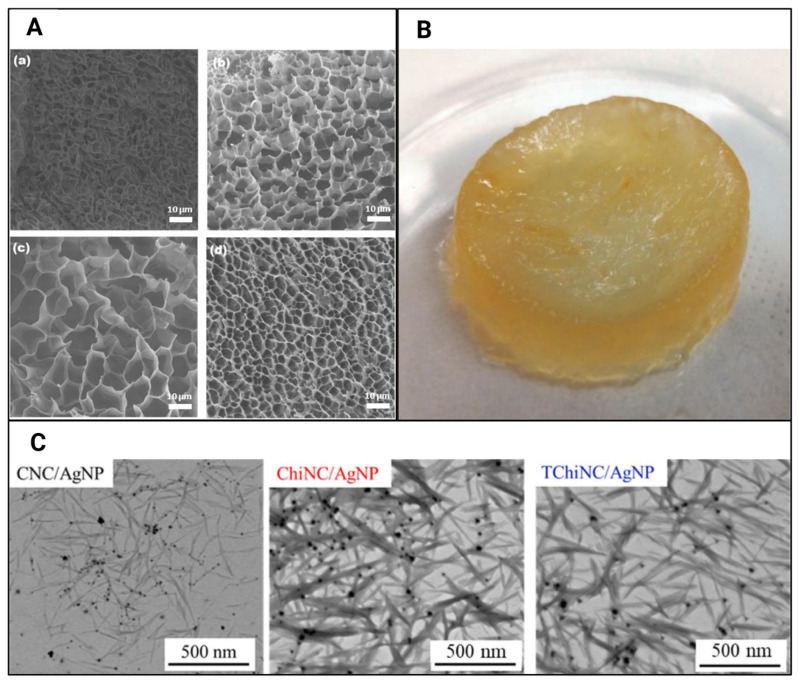
(**A**) Scanning electron microscope (SEM) images of the composite hydrogels (magnification: ×1000) (**a**) net-AAm, (**b**) netAAm/CNC (1% *w*/*w*, (**c**) net-AAm/CNC (2% *w*/*w*), and (**d**) net-AAm/CNC (4% *w*/*w*). Samples irradiated at 15 kGy. Adapted from reference [34]. (**B**) Image of the synthesised hydrogels after the Diels-Alder (DA) reaction. Adapted from reference [31]. (**C**) Scanning transmission electron microscopy (STEM) images of silver nanoparticles (AgNPs) grafted onto various bio-based supports. Adapted from reference [38].

Hydrogels with CNC incorporations have shown promise as primary response materials, particularly in emergencies requiring the dressing of wounds with nosocomial infections. These hydrogels exhibit exudate absorption due to their swelling capabilities [34]. Increasing the CNC concentration in the hydrogel from 1% *w*/*w* to 4% *w*/*w* (Figure 11A) enhanced the ciprofloxacin retention per gram of hydrogel from 1.2 mg to 2.8 mg, attributed to the strong interaction between hydroxyl groups and the drug, thereby improving its loading capacity [34]. Similar trends were observed with silver nanoparticle encapsulation (Figure 11C), where CNC-incorporated hydrogels achieved a 5.3 wt% encapsulation capability compared to 4.2 wt% for TEMPO-oxidised chitin nanocrystal-incorporated hydrogels [38]. The release patterns for the chloramphenicol drug also showed that cumulative drug release increased in hydrogels with higher CNC incorporation (Figure 11B) [31]. Another study reported that ciprofloxacin, primarily retained on the hydrogel surface, was rapidly distributed, with 100% of the drug released within the initial 100 min [34].

##### Antitumour Drug Delivery

Reviewed studies highlight that hydrogels with CNCs have presented themselves as a potential option for advanced tumour treatment [26].

A study developed a KB-cell tumour model in the subcutaneous tissue of female nude mice to investigate the antitumour efficacy of administered doxorubicin-CNC hydrogels. The survival rate of mice increased upon the administration of the hydrogels, signifying the potential of hydrogels in facilitating the chemotherapeutic efficiency of doxorubicin via its sustained release to suppress tumour progression [25].

**Figure 12:**
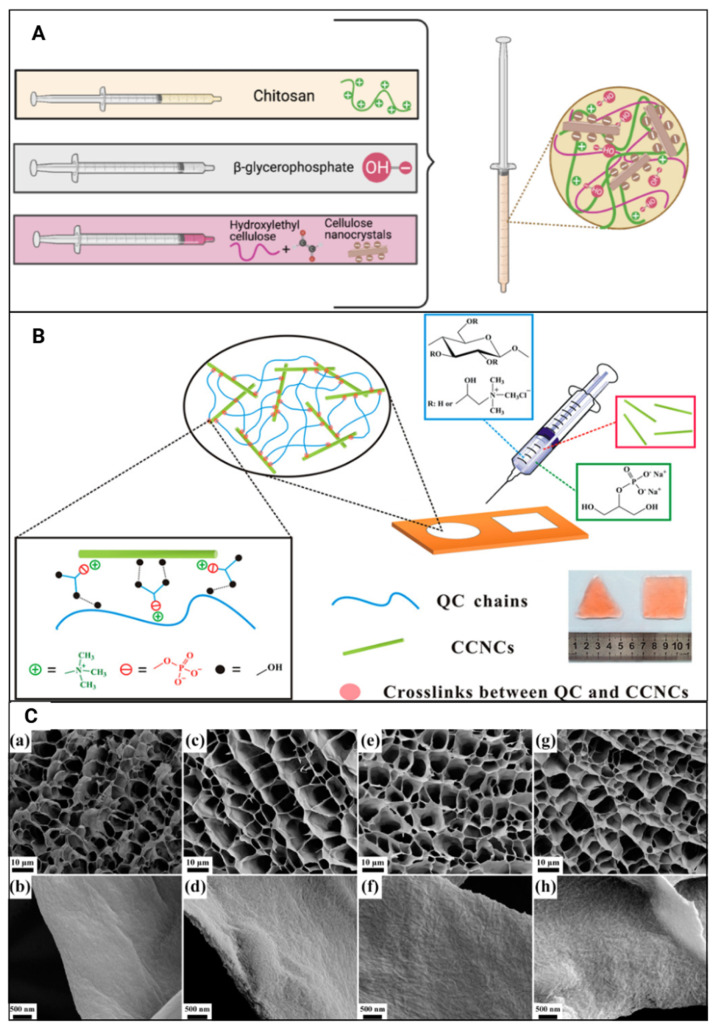
(**A**) Schematic illustration of polymer interactions within the hydrogel matrix. Adapted from reference [42]. (**B**) Schematic representation of hydrogel precursors and injectable QC/CCNC/β-GP nanocomposite hydrogels (Not Drawn to Scale). Adapted from reference [25]. (**C**) Scanning electron microscopy (SEM) images of the cross-section of (**a**,**b**) the pure QC/β-GP hydrogel and the hydrogels reinforced with (**c**,**d**) 1, (**e**,**f**) 1.5 and (**g**,**h**) 2.5 wt% of CCNCs. Adapted from reference [25].

CNC-incorporated hydrogels (Figure 12A) have been utilised in tumoricidal neural stem cell therapy for glioblastoma multiforme [42]. In this study, the cell viability of U87-MG tumour cells was reduced to 66–80% after 24 h and ≥50% after 72 h of exposure to the hydrogels due to the release of therapeutic levels of tumour necrosis factor-α (TNF-α)-related apoptosis-inducing ligand (TRAIL) after 72 h of injection. This was the first study to report the ability of chitosan-CNC injectable hydrogels to promote TRAIL secretion and tumour cell death in vitro [42].

A sustained release of doxorubicin at a rate of 29.4 µg/day over 17 days was reported when utilising CNC-based hydrogels (Figure 12B). The authors noted that this in situ sustained release could enhance antitumour effects by improving the drug’s therapeutic index [25]. The incorporation of CNCs has been reported to progressively increase the viability of C17 mouse neural stem cells (iNSCs) to 98–100% at a concentration of one million cells/mL and 80–84% at concentrations of five and 10 million cells/mL, respectively [42]. CNC-incorporated hydrogels exhibit a slow drug release pattern. One study observed a four- to eight-fold increase in the release of tumour necrosis factor-α (TNF-α)-related apoptosis-inducing ligand (TRAIL) from hydrogels without CNCs [42].

When investigating the effects of CNC-incorporated hydrogels on tumour slices obtained from mice with B16F10 melanoma tumours, the immunofluorescence staining of Ki-67 indicated tumour inhibition with the lack of tumour cell proliferation. Furthermore, a hematoxylin-eosin (H&E) stain of the main organs, including the heart, spleen, lungs and kidney, showed normal cell morphology with no noticeable pathological changes or apoptosis, indicating non-cytotoxicity towards normal cells [26]. Hydrogels with cationic CNCs (Figure 12B) have also been shown to maintain good cell viability in COS-7 cells [25]. Blood compatibility tests were performed on blood samples collected from mice [26] and displayed a lack of red blood cell lysis and negligible toxicity towards blood samples. An AM/PI live/dead assay on the in vitro model displayed cell morphology alterations along with a strong red fluorescence, which confirmed the ability of the hydrogels to kill cancer cells. These results were further supported by the in vivo model displaying tumour growth inhibition without recurrence and a lack of tumour-induced side effects. Therefore, the authors reported these hydrogels as a low-toxic yet effective cancer therapy platform [26].

Hydrogels with 2.5 wt% incorporated CNCs (Figure 12C) displayed 61.7% degradation after 8 days [25]. A 100% biodegradation of hydrogels was observed within a time range of 18–42 days, regardless of CNC incorporation, where the inclusion of higher cell densities was observed to accelerate the degradation rate [42]. Dorsal subcutaneous injections of similar hydrogels in female C57 mice displayed good in vivo biocompatibility due to the progressive reduction in neutrophils and development of tissue similar to healthy tissue at the injection site over 16 days. Over time, the inflammatory responses reduced even further with the degradation of the hydrogels, where any signs of damage, including oedema, hyperaemia, tissue necrosis, muscle damage or haemorrhaging, were not recorded [25].

##### Soft Robot for Controlled Drug Delivery

In the field of biorobotics, one of the breakthroughs is the utilisation of soft robots as a way to navigate through the cellular environment with shape-morphing and adaptable characteristics. Due to the biocompatible nature and favourable mechanical properties of hydrogels, soft robots are being designed using hydrogels for various biomedical applications. Only one of the included studies explored the soft robotic application of CNC-based hydrogels for drug delivery [37], where the details of the study are included in Table 5.

A study reported that CNC-based hydrogels displayed a >95% NIH-3T3 cell viability over 5 days, supporting the biocompatibility of the hydrogels [37]. While CNC-incorporated hydrogels that were not crosslinked displayed fast dissolution of 10 wt% upon immersion, hydrogels that were chemically crosslinked were not fully degradable [37]. Additionally, these hydrogels could be used as patches to navigate drug delivery using magnetism and also pH stimulation [37] to reinforce controlled and sustained drug delivery. This research employed CNCs to create stimuli-responsive hydrogels that operate as soft robots. This functionality arises from CNC’s inherent ability to deform and then return to its original shape, coupled with its pH sensitivity. By aligning CNCs within the hydrogel, the shape-morphing capability can be optimised for efficient deformation and recovery [37]. These soft robots responded to pH changes, transporting and releasing spherical or irregular-shaped soft biological materials when a pH change was detected. The CNC-based soft robot collected cargo in an alkaline environment and released it in an acidic environment. Additionally, a magnetic field could remotely navigate these soft robots to transfer light cargo in confined environments [37]. This highlights the potential of hydrogel-based soft robots for therapeutic drug delivery in alkaline body organ environments due to their pH tolerance.

**Figure 13:**
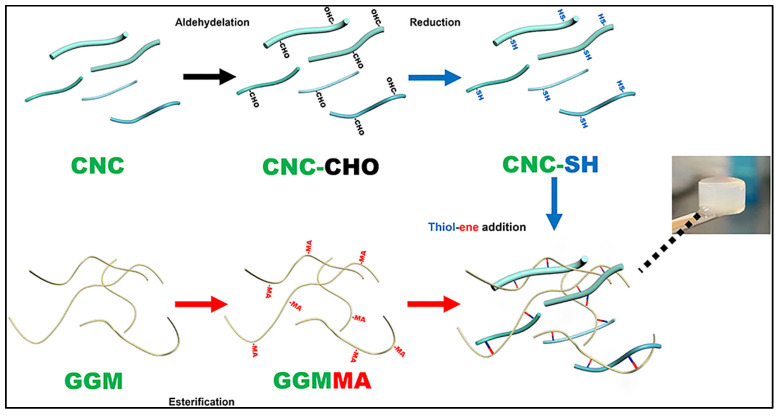
Illustration of hydrogel fabrication. CNCs were isolated from the MCC and oxidised to introduce aldehydes, followed by reductive amination to graft thiol (SH) moieties; galactoglucomannan (GGM) was isolated from the tree, and esterification was performed to introduce methacrylate (MA) moieties. Hydrogel was obtained through light-induced thiol-ene addition. Adapted from reference [23].

When a mannanase-mediated enzymatic degradation was conducted on CNC-based hydrogels (Figure 13), the lowest degradation ratios were observed in hydrogels with 1% thiol-grafted CNC and 1% L-cysteine-grafted oxidised CNC [23]. Sustained release profiles for incorporated silicon, calcium and copper ions were reported, further supporting the controlled and targeted drug delivery over an extended period [23].

##### pH-Controlled Drug Delivery in the Gut

The CNC-incorporated hydrogels have been presented with stomach-specific drug-delivery capabilities as well [33]. A study immersed the CNC-incorporated hydrogels with riboflavin in a solution mimicking the small intestine, as riboflavin absorption mainly occurs in the small intestine. This study observed that osmotic repulsion releases riboflavin molecules due to swelling of the hydrogel, enlarging its pores. Therefore, an initial burst release phase was recorded within the first 0.5–6 h, followed by sustained release over the next 8–12 h. However, the drug release rate slows and the drug release amount reduces due to the congestion of the hydrogel pores, leading to decreased pore size [28]. Drug release patterns mainly followed an initial burst release phase and then a sustained diffusion-controlled release phase [33].

Drugs, such as curcumin [33], when released from hydrogels, were shown to retain the same drug activity as pure drug compounds, as confirmed by the lack of significant differences in UV-vis spectra between their chemical structures. During the initial burst release phase, a higher curcumin concentration gradient was evident due to the retention of molecules on the surface of the hydrogel. However, due to the thickness of the hydrogel acting as a diffusion barrier, curcumin is steadily released for up to 120 min, which indicates enhancement of curcumin bioavailability facilitated by hydrogels [33].

**Figure 14:**
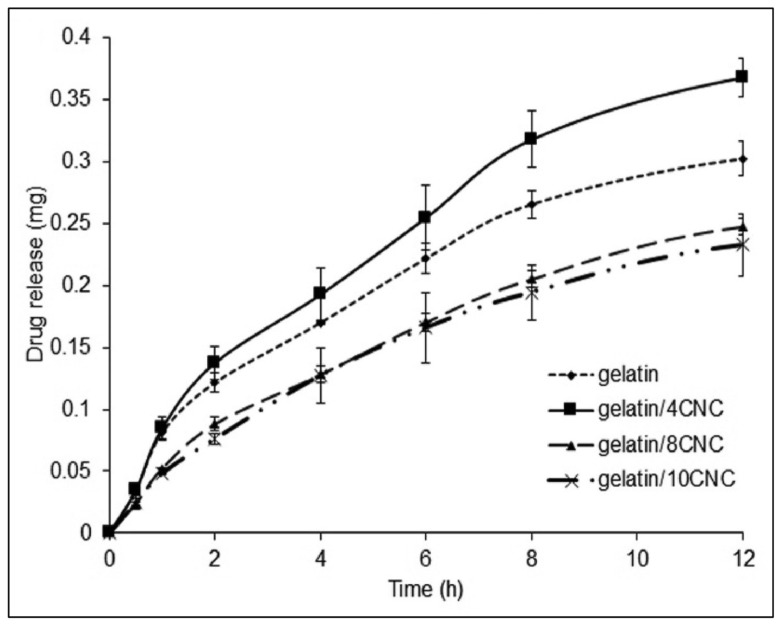
Drug release profiles of hydrogels of the weight of drug release over time. Adapted from reference [28].

Studies used the L929 mouse fibroblast cell line to report that the cell viability increased upon exposure to CNC-based hydrogels [28]. The incorporation of CNCs increases the drug loading capacity since it enhances the swelling capacity of the hydrogel [33], which was seen when loaded with riboflavin (10 mg/mL) [28]. However, riboflavin loading efficiency was gradually reduced as the CNC content was >4% (Figure 14) [28] due to the prevention of drug penetration through the rigid structure of the hydrogel that resulted from CNCs filling the voids within the porous structure of the hydrogel [33].

**Figure 15:**
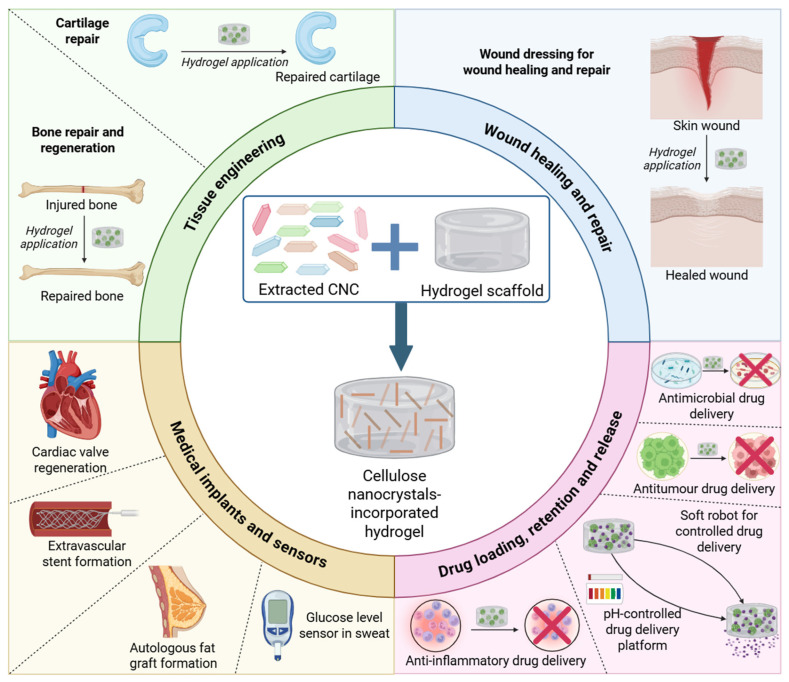
Development of a cellulose nanocrystal (CNC)-incorporated hydrogel for emerging biomedical applications (created by D.M. Seneviratne using BioRender (https://app.biorender.com/ accessed on 29 April 2025).

Overall, the included studies in this scoping review highlighted the methods utilised to extract CNCs from sources such as wood pulp, Whatman filter paper, rice husk and corkboard, which were then incorporated into hydrogels for a multitude of emerging biomedical applications. These applications of CNC-incorporated hydrogels, along with CNC sources and extraction methods, are summarised in Figure 15.

## 3. Conclusions

Cellulose nanocrystal (CNC)-incorporated hydrogels have emerged as promising candidates for various biomedical applications due to their biocompatibility, sustainability, and tunable characteristics. This scoping review has examined the primary research studies published to date that have reported on the addition of CNCs to hydrogels that could be used in biomedical and/or healthcare applications. The studies included in this review have investigated CNC hydrogels for applications in hard and soft tissue engineering, wound healing and repair, medical implants and sensors, and drug delivery, including antimicrobials for infection control and chemotherapy drugs for cancer treatment. This analysis revealed that the majority of CNCs were sourced from microcrystalline cellulose, the extraction methods included sulfuric acid hydrolysis and TEMPO-oxidisation, and the modifications ranged from silver nanoparticle and thiol grafting to glucose oxidase loading, TEMPO oxidation, magnetic CNCs, dialdehyde CNCs, cationic CNCs, and tunicate CNCs. While the reviewed CNC-based hydrogels demonstrated favourable characteristics over their non-CNC control hydrogels, future studies should focus on advancing this work to human clinical trials and optimising CNC hydrogel formulations to fully realise their potential in the proposed biomedical and healthcare applications.

## 4. Methodology

### 4.1. Data Sources and Searches

A literature search was performed in November 2024 to identify English-language (and translated), peer-reviewed primary research articles that were indexed in PubMed Central, PubMed, BioMed Central, ScienceDirect, Wiley, and EBSCOhost databases. The Preferred Reporting Items for Systematic Reviews and Meta-Analyses (PRISMA) guidelines were followed when developing the search protocol. The databases were systematically searched using the following Boolean search terms: (Functional properties OR properties) AND hydrogel* AND (“cellulose nanocrystals” OR nanocellulose OR nanowhiskers) AND (healthcare OR “healthcare applications” OR biomedicine OR biomedical OR “biomedical applications”). It should be noted that there were some variations in the search approach in the selected databases to suit the search platform.

### 4.2. Inclusion and Exclusion Criteria

The current study included peer-reviewed publications that satisfied the following criteria:

- Open access and/or open archive availability of the full-text article
- Publication type is a journal article
- Primary research study design
- Applications to healthcare or biomedicine
- Utilisation of cellulose nanocrystals or nanocellulose, or nanowhiskers
- Utilisation of appropriate controls, including negative (hydrogel with no CNC incorporation) and positive controls

Any studies that were not primary research articles investigated the effects of bacterial nanocellulose- and cellulose nanofiber-based hydrogels, and no healthcare or biomedical applications were excluded.

### 4.3. Study Selection and Quality Assurance

Two authors screened the included studies in two stages: 1. Title and abstract screening to identify the eligibility of the articles by reading the titles and abstracts, and 2. Full-text screening further streamlined the inclusion of the articles using the Joanna Briggs Institute System for the Unified Management of the Assessment and Review of Information (JBI SUMARI), where a third author resolved any conflicts during these stages after the full articles were read. A thematic analysis using the Xmind (Version 24.04.10311) mind-mapping software was conducted to identify the main themes that were investigated in the included articles.

## Figures and Tables

**Table 1:** CNC sources, extraction, and modifications of included studies.

Reference	CNC Source	CNC Extraction Method	CNC Modifications
[23]	Microcrystalline cellulose (MCC; Avicel PH-101; Fluka)	Sulphuric acid hydrolysis	Thiol-grafted CNC (CNC-SH); L-cysteine-grafted oxidised CNC
[24]	MCC (Tianjin Komeo Chemical Reagent)		Glucose oxidase (Gox)-loaded CNC using CNCs@BSA-Gox (CBG)
[25]	MCC with a degree of polymerisation of 100–300 (Asahi Kasei Corporation, Japan)		Cationic CNC (CCNC)
[26]	Cotton (from a local retailer, Shanghai, China)		Dialdehyde CNC
[27]	Cotton cellulose from Whatman ashless filter-aid paper (Sigma-Aldrich)		-
[28]	Rice husk (Bernas Sdn. Bhd., Shah Alam, Selangor, Malaysia)		
[29]	MCC (manufacturer details not provided)		
[30]	MCC (Sigma-Aldrich)		
[31]			
[32]	Microcrystalline fibrous cellulose powder from Whatman-CF11 filter paper (Sigma-Aldrich Co.)		
[33]	MCC (R&M Chemicals, Essex, UK)		
[34]	MCC (Avicel PH-101, particle size of 50 µm; Sigma-Aldrich, Toluca, Mexico)		
[35]	α-cellulose (Sigma-Aldrich)		
[36]	Commercially purchased (solid content of 6%, Cellulose Lab)	-	TEMPO-oxidised CNC
[37]	Commercially purchased (CelluForce Inc., Canada)		Magnetic CNC; shear-induced preferential CNC alignment
[38]	Commercially purchased (product number 2015–009, CelluForce, Canada)		Silver nanoparticle-CNC hybrid suspensions via dissolution of AgNO_3_ in CNC
[39]	Commercially purchased (CELLUFORCE NCV100–NASD90; CelluForce, Montreal, QC, Canada)		-
[40]	Commercially purchased (particle size < 150 nm, CELLUFORCE NCV100—NASD90, Windsor, ON, Canada)		
[41]	Corkboard	TEMPO-oxidation of corkboard and mild disintegration in water	-
[42]	Not provided	-	-
[43]			Tunicate CNC (TCNC)
[44]	Commercially purchased (enzymatically pretreated tunicate nanocellulose; Ocean TuniCell AS, Blomsterdalen, Norway)	-	

**Table 2:** Bone and cartilage tissue engineering applications of cellulose nanocrystal (CNC)-based hydrogels.

Reference	Components of the Hydrogel	Influence of CNC	Biomedical Applications
[27]	Chitosan, CNCs	Increased viscosity of hydrogel bioinks; promoted osteogenesis onset, collagen formation in extracellular matrix (ECM) and calcium deposition; reinforcing agent of the hydrogel; no impact on cell viability or proliferation of clonal non-transformed newborn mouse calvaria MC3T3-E1 pre-osteoblast cartilage-like cells	Bone tissue engineering and bone defect repairing
[43]	Chitosan, CNCs	Increased proliferation and survival of clonal non-transformed newborn mouse calvaria MC3T3-E1 pre-osteoblast cartilage-like cells; increased osteogenic differentiation of MC3T3-E1 cells, collagen formation in ECM and calcium deposition	Bone tissue engineering
[29]	Sodium alginate, sericin, CNCs	Supported clonal non-transformed newborn mouse calvaria MC3T3-E1 pre-osteoblast cartilage-like cell survival and proliferation; diminished biodegradation via strong crosslinking; promoted osteogenic differentiation and biomineralisation capacity	
[35]	MeGel, hyaluronic acid methacrylate (HAMA), CNCs, lithium phenyl 2,4,6-trimethyl-benzoylphosphinate (LAP)	Increased chondrogenic ATDC5 cell proliferation	Cartilage tissue repair and regeneration
[39]	Alginate, CNCs	Improved human-derived TC28a2 immortalised chondrocyte viability, especially in bioprinted hydrogels and maintenance of cell morphology	Bioink formulation for the fabrication of cartilage soft tissue

**Table 3:** Wound healing and repair capabilities of cellulose nanocrystal (CNC)-based hydrogels.

Reference	Components of the Hydrogel	Influence of CNCs (Compared to No-CNC Hydrogel Control)	Biomedical Applications
[30]	CNCs, pectin, chitosan	Increased adult human dermal fibroblast cell numbers over seven days for 10% CNC; increased antibacterial activity against *Staphylococcus aureus* and *Escherichia coli* at 10% and 20% CNCs (Figure 6B)	Wound healing and antimicrobial activity

**Table 4:** Outline of medical implant and biosensor applications of cellulose nanocrystal (CNC)-based hydrogels.

Reference	Components of the Hydrogel	Influence of CNC	Biomedical Applications
[36]	Methacrylated-gelatine (MeGel), TEMPO-oxidised CNC	Improved stiffness of the hydrogel via intra- and intermolecular hydrogen bonds; promoted human adipose-derived mesenchymal stem cell migration and metabolic activity; increased GAG deposition; decreased hydroxyproline content and calcification; inhibited osteogenic differentiation of human adipose-derived mesenchymal stem cells; reduced SMA and MMP2 expressions and increased vimentin expression to promote a fibroblastic phenotype of human adipose-derived mesenchymal stem cells	Cardiac valve engineering
[41]	CNCs, gelatine, genipin, astragaloside IV (AS-IV)	Good human umbilical vein endothelial cell proliferation and local tissue compatibility with no significant impact on important organs and inflammatory responses; controlled biodegradation and drug release	Extravascular stents inhibiting restenosis via preventing endothelial-to-mesenchymal transition (EndMT) after coronary artery bypass grafting and haemodialysis access; Drug delivery of astragaloside IV (AS-IV)
[44]	Lipoaspirate adipose (LAT) fraction, enzymatically pretreated tunicate nanocellulose (ETC), sodium alginate, CaCO3 microparticles (CMP), Glucono-δ-lactone (gluconolactone; GDL)	Assistance in shape and volume retention in grafts, resulting in retention of more adipocytes	Volume and shape retention and distribution of adipose cells in autologous fat-hydrogel grafts
[24]	PVA, glucose oxidase (Gox)-loaded CNC using BSA, iron (III) ions	Carrier of BSA and Gox via adsorption and surface grafting, respectively; no significant effect on adherence properties of the hydrogel or the current response to various glucose concentrations	Glucose level sensor in human sweat

**Table 5:** Drug loading, retention, and release capacities of cellulose nanocrystal (CNC)-based hydrogels.

Reference	Components of the Hydrogel	Influence of CNC	Biomedical Applications
[32]	Aminopropyl-functionalised magnesium phyllosilicate (Organoclay), CNC	Physical immobilisation and controlled release of the drug	Anti-inflammatory drug, ibuprofen, loading and releasing
[40]	Poly(2-(dimethylamino)ethyl methacrylate) (PDMAEMA), CNC	No effect on drug absorption or altering the thermal degradation of hydrogels; Improved encapsulation efficiency of drugs and controlled drug release pattern	
[31]	Gelatinised starch (S) with furfuryl isocyanate (FI) (S-FI), CNCs, tetra maleimide (TTMI), chloramphenicol	Improved mouse L929 fibroblast viability and drug loading; controlled drug release	Antimicrobial drug, chloramphenicol, loading and releasing
[34]	Aqueous solutions of acrylamide (net-AAm), CNC	Increased drug loading and retention capabilities as the CNC content increases	Antibiotic drug, ciprofloxacin, loading and releasing with potential for wound healing and treating infections
[38]	Calcium alginate, silver nanoparticles, CNCs, or TEMPO-oxidised chitin nanocrystals	Improved silver nanoparticle encapsulation and retention; a biopolymeric template for grafting silver nanoparticles and stabilising them	Antifungal drug, silver, encapsulation and release
[42]	Chitosan, CNC	Reinforcing agent; improved gelation kinetics; prolonged hydrogel degradation; assisted in sustained release of therapeutic C17 mouse neural stem cells from hydrogels with no significant difference in cell viability; controlled release of tumour necrosis factor-α (TNF-α)-related apoptosis-inducing ligand (TRAIL) protein	Neural stem cell-laden TRAIL proapoptotic agent-incorporated hydrogel for post-surgical glioblastoma multiforme treatment
[25]	Quaternised cellulose (QC), CNCs, β-glycerophosphate (β-GP)	Initially triggered inflammatory responses gradually diminished; no evidence of necrosis, haemorrhaging, oedema, or muscle damage; improved dimensional stability; slow degradation rate; controlled drug release	Localised and sustained antitumour drug, doxorubicin, delivery
[26]	CCHO, nano carbon dots	High biosafety and negligible cytotoxicity as evaluated on mouse B16F10 melanoma cells and HeLa cervical cancer cells; irradiated hydrogels have tumour-killing ability towards B16F10 tumour-bearing nude mice through simultaneous photothermal therapy and photodynamic therapy	Injectable tumour therapy platform
[37]	CNCs or magnetic CNCs with and without preferential alignment, 3-dimethyl (methacryloyloxyethyl) ammonium propane sulfonate (DMAPS, 95%), methacrylic acid (MAA, 99%)	Magnetic CNC: Magnetic navigation of the soft robot using superparamagnetic behaviour; increased mouse NIH-3T3 cell viability and proliferation; controlled biodegradation	Soft robot for grabbing, moving, and releasing soft and light biological cargo
		Shear-induced preferential CNC alignment: Inducing structural anisotropy, application of shear leads to shear thinning, reorientation, and alignment of the hydrogel	
[23]	Methacrylated-galactoglucomannan (GGMMA); CNC-SH or L-cysteine-grafted oxidised CNC; bioactive glass nanoparticle (BaGNP); Copper-BaGNP	Tailor degradation of hydrogels; localised and controlled drug delivery	Extended therapeutic release of silicon and calcium ions for wound healing
[28]	CNCs, gelatine, riboflavin	Good biocompatibility with slight toxicity towards mouse L929 fibroblast cells; inverse proportionality between CNC content and drug loading and releasing	Drug loading and release in the gut
[33]	CNCs, chitosan, curcumin	Enhancing drug loading capacity; controlled biodegradability and drug release capability at the appropriate site to enhance bioavailability of stomach and upper intestinal tract-related drugs	Stomach and upper intestinal tract-related drug, curcumin, loading and releasing

## Data Availability

No new data were created or analysed in this study.
